# Prevalence of HBsAg, knowledge, and vaccination practice against viral hepatitis B infection among doctors and nurses in a secondary health care facility in Lagos state, South-western Nigeria

**DOI:** 10.11604/pamj.2016.23.160.8710

**Published:** 2016-04-06

**Authors:** Abdul-Hakeem Olatunji Abiola, Adebukola Bola Agunbiade, Kabir Bolarinwa Badmos, Adenike Olufunmilayo Lesi, Abdulrazzaq Oluwagbemiga Lawal, Quadri Olatunji Alli

**Affiliations:** 1 Department of Community Health and Primary Care, College of Medicine of the University of Lagos, Lagos, Nigeria; 2 General Hospital Lagos, Lagos, Nigeria; 3 Department of Morbid Anatomy, College of Medicine of the University of Lagos, Lagos, Nigeria; 4 Department of Medicine, College of Medicine of the University of Lagos, Lagos, Nigeria; 5 Department of Surgery, College of Medicine of the University of Lagos, Lagos, Nigeria; 6 Island Maternity Hospital Lagos, Lagos, Nigeria

**Keywords:** hepatitis B surface antigen, hepatitis B vaccination, health

## Abstract

**Introduction:**

Hepatitis B Virus, a highly infectious blood-borne virus poses a major threat to public health globally due to its high prevalence rate and grave consequence in causing liver cirrhosis and hepatocelullar carcinoma, the third cause of cancer death worldwide. The aim is determine the prevalence of HBsAg, knowledge, and vaccination practices against viral hepatitis B infection among doctors and nurses in a health care facility.

**Methods:**

Study design was a descriptive cross-sectional study among all the doctors and nurses in the health care facility. Data was collected using pre-tested, structured, self-administered questionnaire and blood samples were taken from respondents and tested using commercial enzyme-linked immunosorbent assay (ELIZA) test kit to determine prevalence of hepatitis B surface antigen after informed consent. Ethical approval was obtained from Health Research and Ethics Committee of the Lagos University Teaching Hospital. Responses of the respondents to the knowledge and vaccination practices against viral hepatitis B infection were scored and graded as poor (<50%), fair (50-74%) and good (≥75%). The study was carried out in January, 2014.

**Results:**

A total of 134 out of the 143 recruited respondents participated in the study. Prevalence of HBsAg was 1.5%. Among the respondents, 56.7% had good knowledge and 94.8% reported poor practice of vaccination against viral hepatitis B infection. Mean knowledge and vaccination practices scores (%) were 72.54+7.60 and 29.44+14.37 respectively. Only 29% of the respondents did post vaccination testing for anti HBsAg.

**Conclusion:**

Prevalence of HBsAg was low. Knowledge of viral hepatitis B was fair, and practice of post hepatitis B vaccination testing was poor. It is therefore recommended that the state ministry of health should organise further health education programme, institute compulsory occupational hepatitis B vaccination programme and post vaccination anti-HBS testing to ensure adequate antibody level in this adult population.

## Introduction

HBV, a highly infectious blood-borne virus, is responsible for about 80% of all cases of hepatocelullar carcinoma, which is the third leading cause of cancer death in Asia and Africa [1]. Hepatitis B Virus has been estimated to infect more than 2 billion people globally due to its endemicity; 350million of these people are chronically infected and become carriers of the virus and 10-30million will become infected annually. An estimate of more than 1million die annually, while approximately 2 people die each minute from HBV infection [2–5]. About a quarter of the chronic carriers will later die from hepatic complications. While some remain life-long carriers, others will clear the infection after varying interval [2, 6]. The prevalence of HBV infection varies, depending on a number of factors which includes the geographical region, host factors as well as other environmental/behavioural factors. A larger percentage of areas of North America have a low prevalence (<2%). This low prevalence could be attributed to the high standard of living in these areas, although some high prevalence pockets exist, particularly in areas with a high proportions of Asian immigrants, Alaskan and northern Canadian native populations, where rates as high as 5% - 15% of chronic HBV or prevalence of HbsAg positivity are found [3, 7]. The acquisition of HBV infection in most of these low prevalence regions occur mainly during adolescence and mid-adulthood [2]. India has intermediate endemicity of Hepatitis B with a prevalence of hepatitis B surface antigen (HBsAg) between 2% and 7%. The number of HBsAg carriers in India has been estimated to be over 50 millions [1]. Studies done in Nigeria showed HBV carriage rate in the range of 9-39% [8]. In a study done among surgeons in Nigeria, the prevalence of HBsAg among the doctors (surgeons) was 25.7%, the prevalence of anti-HBs was 22.2% in the doctors and that of antiHBc was 61.7% in the doctors, while no HBV marker was detected in 23.4% of the doctors [9]. HBV is a priority occupationally acquired infection that is associated with serious public and personal health consequences, and is considered to be the most important cause of occupational acquired viral hepatitis amongst doctors and nurses who are particularly exposed via contact with blood and secretions in the course of discharging their duty [3, 4]. The risk of acquiring HBV in these cadres of health workers is four times greater than that of the general population [10]. An estimate of about 600,000-800,000 cuts and puncture injuries occur in them annually, out of which approximately 50% are not registered [11]. Hepatitis B is a vaccine preventable disease, though a sizeable proportion of HCWs never get vaccinated. Both the pre-exposure and post-exposure administration of the vaccines have been recommended [2, 12]. This has been a major reason for advocating compulsory vaccination against HBV among health care workers before being exposed to patients to prevent serious consequences including long term illness, debility and death [13]. Therefore, doctors and nurses should be aware of the risks involved in treatment procedures, and should endeavour to take due precautions in dealing with patients, since knowing facts and having proper attitudes are very crucial to prevent the prevalence and spread of HBV, or its reduction to the barest minimum [11]. This study was therefore carried out to determine the prevalence of HBsAg, knowledge and vaccination practices against viral hepatitis B infection among doctors and nurses in Lagos Island Maternity Hospital.

## Methods

**Study Location:** the study was carried out in Lagos Island Maternity Hospital, located in Lagos Island, an urban community of Lagos state in South Western, Nigeria. The Hospital, a 500-bed secondary health care facility for Obstetrics and Gynaecology cases, served as a referral centre to other health centres and other general hospitals from neighbouring towns both within and outside Lagos state. The hospital benefited from the occupational hepatitis B vaccination programme carried out by the Lagos state government [14]. There were 48 doctors and 95 nurses in the hospital at the time of the study.

**Methods:** the study design was a descriptive cross-sectional study among doctors and nurses in Lagos Island Maternity Hospital. With a confidence interval of 95%, degree of accuracy desired of 5%, and prevalence of HBsAg in a previous study [9] of 25.7%, the minimum sample size of 107 was determined using the formula for descriptive cross-sectional study when sampled population is less than 10,000. However, all the 143 doctors and nurses (48 doctors and 95 nurses) in the hospital were enrolled into the study. The study was carried out in January 2014. A pre-tested, structured, self-administered questionnaire which was derived from other similar published studies [15–17] was used to collect information on socio-demographic characteristics, knowledge of HBV infection and practice of hepatitis B vaccination. Each correct response to the knowledge and vaccination practice questions was scored one mark and any wrong or non response was scored zero. The total score obtained by each respondent was converted to percentage and graded as poor (<50%), fair (50-74%) and good (≥75%). The mean knowledge and practice scores (%) for all the respondents were also calculated. Laboratory technique using commercial enzyme-linked immunosorbent assay (ELIZA) kits was used to determine prevalence of hepatitis B surface antigen. Sample with cut-off index greater than or equal to 1.0 was considered positive for HBsAg.

**Data analysis:** data analysis was done using Epi-info version 3.5.1, WinPepi and GraphPad Instat statistical software packages. Chi-square and Fisher's exact tests were used to compare differences between proportions while t-test and analysis of variance (ANOVA) were used to compare differences between means. P value ≤0.05 was considered statistically significant.

**Ethical Issues:** ethical approval was obtained from Health Research and Ethics Committee (HREC) of the Lagos University Teaching Hospital (LUTH). Verbal informed consent was obtained from the respondents.

## Results

**Response rate:** a total of 134 out of the 143 doctors and nurses participated in the study, giving a response rate of 93.7%. Socio-demographic characteristics of the respondents: The mean age of the respondents was 35.86+8.61 years. Majority of the respondents were females (72.39%), Christians (74.63%), nurses (64.93%), unmarried (59.70%) and had spent less than 10years on their current job (82.84%) (Table 1).

**Table 1:** Distribution of respondents by sociodemographic characteristics according to hepatitis B Serostatus

Socio-demographic	Hepatitis B Serostatus Frequency (%)			Statistics and Pvalue
Characteristics	Negative	Positive	Total	
**Age-group (years)**				
20-29	41(31.06)	0 (0.00)	41(30.60)	
30-39	50(37.88)	1 (50.00)	51(38.06)	
40-49	34(25.76)	1 (50.00))	35(26.12)	
50-59	7(5.30)	0(0.00)	7(5.22)	
Total	132(100.00)	2(100.00)	134(100.00)	
Mean±SD	35.81±8.63	39.00±8.49	35.86 + 8.61	t=0.52; df= 132; p=0.605
**Sex**				
Male	36(27.27)	1(50.00)	37(27.61)	**X^2^=0.509**
Female	96(72.3)	1(50.00)	97(72.39)	df=1
Total	132(100.00)	2(100.00)	134(100.00)	p=0.098
**Occupation**				
Doctors	46(34.85)	1(50.00)	47(35.07)	**X^2^=0.199**
Nurses	86(65.15)	1(50.00)	87(64.93)	df=1
Total	132(100.00)	2(100.00)	134(100.00)	p=0.656
**Religion**				
Christianity	98(74.24)	2(100.00)	100(74.63)	**X^2^=0.69**
Islam	34(25.76)	0(0.00)	34(25.37)	df=1
Total	132(100.00)	2(100.00)	134(100.00)	p=0.406
**Marital status**				
Not married	80(60.61)	0(0.00)	80(59.70)	**X^2^=3.0079**
Married	52(39.39)	2(100.00)	54(40.30)	df=1
Total	132(100.00)	2(100.00)	134(100.00)	p=0.083
**Years of experience**				
Less than 10yrs	110(83.33)	1(50.00)	111(82.84)	
10yrs and above	22(16.67)	1(50.00)	23(17.16)	
Total	132(100.00)	2(100.00)	134(100.00)	
Mean±SD	5.01±5.20	12.50 ±16.26		t=1.96; df=132; p=0.052

**Prevalence of HbsAg:** only 2(1.5%) of the respondents were found positive for HBsAg. Among the respondents who were HBsAg seropositive, 1(50.0%) was a male, and 1(50.0%) was a female; 1(50.0%) was a nurse and 1(50.0%) was a doctor by profeesion; the 2 were Christians (100.0%), married (100.0%) and 1(50.0%) had spent less than 10 years on current job. There was no statistically significant relationship (p>0.05) between the sociodemographic characteristics and viral hepatitis B Serostatus (Table 1). Knowledge of viral Hepatitis B: Majority of the respondents (56.70%) had good knowledge while 43.30% had fair knowledge of hepatitis B infection although, there were observed gaps: Over a third (34.3%) of the respondents knew that a person who has recovered from viral hepatitis B does not transmit the virus; only 32.8% of the respondents knew that hepatitis B vaccine has no effect on HBsAg carriers; and only 30.6% of the respondents knew that both pre-exposure and post-exposure administration of hepatitis B vaccine is recommended. The mean knowledge score (%) which suggests the depth of knowledge on the aspects of knowledge investigated was 72.54+7.60 (Table 2). There was no statistically significant relationship (p>0.05) between the sociodemographic characteristics and knowledge of viral hepatitis B infection (Table 3).

**Table 2:** Respondents’ knowledge of viral hepatitis B infection

Knowledge of Viral Hepatitis B infection	Correct Response Frequency (%) n=134
**Prevention and containment**	
**Hepatitis B vaccine**	
Three doses of Hepatitis B vaccine are required for complete protection	110(82.1)
An effective antibody response is generally attained after 3 doses in 95 percent of vaccinees.	100(74.6)
First dose is given at elected date, second dose is given 1 month after 1^st^ dose while 3rd dose is given 6 months after the 1st dose	88(65.7)
Immunity continues at protective levels for approximately 3-5 years after vaccination	73(54.5)
Patients who are vaccinated against hepatitis B should not be considered as a possible source of hepatitis B infection	68(50.7)
Hepatitis B vaccine has no effect on HBsAg carriers	44(32.8)
Both pre-exposure and post-exposure administration of hepatitis B vaccine is recommended	41(30.6)
** • Other measures**	•
Proper needles/sharps disposal can prevent viral Hepatitis B	122(91.0)
All blood donors should be screened for HBV infection and those positive for Australian antigen should be rejected	129(96.3)
**Knowledge Grade**	
Poor	0(0.0)
Fair	58(43.3)
Good	76(56.7)
Total	134(100.0)
**Mean knowledge score (%)**	**72.54±7.60**

**Table 3:** Association between socio-demographic characteristics and Knowledge of viral hepatitis B

Socio-demographic	Knowledge of viral hepatitis B Frequency (%)		Statistics and Pvalue
Characteristics	Fair	Good	
**Age-group (years)**			
20-29	21(51.2)	20(48.8)	
30-39	20(39.2)	31(60.8)	
40-49	14(40.0)	21(60.0)	
50-59	3(42.9)	4(57.1)	
Total	58(43.3)	76(56.7)	
Mean±SD	34.86±9.05	36.62±8.23	t=1.77; df= 132; p=0.07
**Sex**			
Male	18(48.7)	19(51.3)	
Female	40(41.24)	57(58.8)	X^2^ =0.599; df=1; p=0.438
Total	58(43.3)	76(56.7)	
**Occupation**			
Doctors	20(42.6)	27(57.4)	
Nurses	38(43.7)	49(56.3)	X^2^ =0.016; df=1; p=0.900
Total	58(43.3)	76(56.7)	
**Religion**			
Christianity	44(44.0)	56(56.0)	
Islam	14(41.2)	20(58.8)	X^2^ =0.082; df=1; p=0.774
Total	58(43.3)	76(56.7)	
**Marital status**			
Not married	38(47.5)	42(52.5)	
Married	20(37.0)	34(63.0)	X^2^ =1.043; df=1; p=0.307
Total	58(43.3)	76(56.7)	
**Years of experience**			
Less than 10yrs	49(44.1)	62(55.9)	
10yrs and above	9(39.1)	14(60.9)	
Total	58(43.3)	76(56.7)	
Mean±SD	4.16±5.00	5.86 ±5.65	t=1.82; df=132; p=0.071

**Practice of hepatitis B vaccination:** Majority (94.8%) of the respondents reported poor practice while 5.2% reported fair practice of hepatitis B vaccination: 22.4% did not receive any dose of hepatitis B vaccine, 11.9% received 1 dose of hepatitis B vaccine, 17.2% received 2 doses of hepatitis B vaccine, and 48.5% received the complete 3 doses of hepatitis B vaccine. About one-third (29%) of the respondents checked their immunity against hepatitis B after vaccination, while only 23.9% of the respondents found out they were protected after receiving the complete 3 doses of hepatitis B vaccine. Respondents also gave reasons for not being vaccinated: 0.7% said that the vaccine was too expensive, 1.5% said the vaccine was not available, 0.7% said they were scared because the vaccine hurts while 19.4% gave no reason. The mean practice score (%) which suggests the depth of practice of hepatitis B vaccination on the aspects of practice investigated was 29.4+14.4. There was statistically significant relationship (p=0.046) between occupation of respondents and practice of hepatitis B vaccination (Table 4). There was also no statistically significant relationship (p>0.05) between any of the socio-demographic characteristics and number of doses of hepatitis B vaccine received (Table 5).

**Table 4:** Association between socio-demographic characteristics and Practice of hepatitis B vaccination

Socio-demographic	Practice of hepatitis B vaccination Frequency (%)		Statistics and P value
Characteristics	Poor	Fair	
**Age-group (years)**			
20-29	38 (92.7)	3(7.3)	
30-39	48 (94.1)	3(5.9)	
40-49	34 (97.1)	1(2.9)	
50-59	7 (100.0)	0(0.0)	
Total	127(94.8)	7(5.2)	
Mean±SD	36.17±8.52	30.29±8.86	t=1.77; df= 132; p=0.07
**Sex**			
Male	37 (100.0)	0(0.0)	
Female	90 (92.8)	7(7.2)	X^2^ =2.817; df=1; p=0.093
Total	127(94.8)	7(5.2)	
**Occupation**			
Doctors	47 (100.0)	0(0.0)	
Nurses	80 (92.0)	7(8.0)	X^2^ =3.99; df=1; p=0.046
Total	127(94.8)	7(5.2)	
**Religion**			
Christianity	93 (93.0)	7(7.0)	
Islam	34 (100.0)	0(0.0)	X^2^ =2.51; df=1; p=0.113
Total	127(94.8)	7(5.2)	
**Marital status**			
Not married	74 (92.5)	6(8.0)	
Married	53 (98.1)	1(1.9)	*p=0.24
Total	127(94.8)	7(5.2)	
**Years of experience**			
10yrs and above	22 (95.7)	1(4.3)	
Less than 10yrs	105(94.6)	6(5.4)	
Total	127(94.8)	7(5.2)	
Mean±SD	5.2 ±5.49	3.5±4.27	t=0.81; df=132; p=0.42

**Table 5:** Association between Socio-demographic characteristics and number of doses of hepatitis B vaccine received

Sociodemographic	Vaccine Doses				Statistics
Characteristic	0 Dose	1 Dose	2 Doses	3 Doses	and P value
**Age-grp(yrs)**					
20-29	4 (9.8)	9 (22.0)	9 (22.0)	19(46.3)	
30-39	16(31.4)	4 (7.8)	9 (17.6)	22(43.1)	
40-49	9 (25.7)	2 (5.7)	5 (14.3)	19(54.3)	
50-59	1 (14.3)	1 (14.3)	0 (0.0)	5 (71.4)	
Total	30(22.4)	16(11.9)	23(17.2)	65(48.5)	
Mean±SD	37.97±7.54	32.00±7.62	38.83±7.58	36.55±9.33	p=0.08 (ANOVA)
**Sex**					
Male	8 (21.6)	4(10.8)	8 (21.6)	17(45.9)	x^2^=0.73
Female	22(22.7)	12(12.4)	15(15.5)	48(49.5)	df=3
Total	30(22.4)	16(11.9)	23(17.2)	65(48.5)	p=0.87
**Religion**					
Christianity	24(24.0)	8(8.0)	16(16.0)	52(52.0)	**x** ^2^=6.88
Islam	6(17.6)	8(23.5)	7(20.6)	13(38.2)	df=3
Total	30(22.4)	16(11.9)	23(17.2)	65(48.5)	p=0.08
**Occupation**					
Doctor	8(17.0)	7(14.9)	13(27.7)	19(40.4)	x^2^=7.08
Nurse	22(25.3)	9(10.3)	10(11.5)	46(52.9)	df=3
Total	30(22.4)	16(11.9)	23(17.2)	65(48.5)	p=0.07
**Marital Status**					
Not married	16(20.0)	10(12.5)	14(17.5)	40(50.0)	x^2^ **=**0.66
Married	14(25.9)	6 (11.1)	9 (16.7)	25(46.3)	df=3
Total	30(22.4)	16(11.9)	23(17.2)	65(48.5)	p=0.88
**Years of experience**					
Less than 10yrs	22(19.8)	15(13.5)	20(18.0)	54(48.6)	
10yrs and above	8(34.8)	1(4.3)	3(13.0)	11(47.8)	
Total	30(22.4)	16(11.9)	23(17.2)	65(48.5)	
Mean±SD	6.98±6.04	4.22±5.72	4.46±4.55	4.72±5.27	p=0.20 (ANOVA)

## Discussion

**Prevalence of Hepatitis B Surface Antigen:** The finding that only 1.5% of the respondents were positive for HBsAg is lower than the findings of 25.7% among surgeons in a study carried out in Lagos [9] but is similar to the findings of 2.18% and 2.4% from the studies conducted in Peshawar, Pakistan [18] and Korea [19] respectively. The low prevalence of HBsAg in this study may be attributed to the increased awareness of viral Hepatitis B infection and high prevalence of prior vaccination in the subjects. Furthermore, since screening for HBSAg is done before any vaccination is commenced; those who were seropositive might have been transferred to administrative units in the state ministry of health.

**Knowledge of Viral Hepatitis B:** The finding that all the respondents had good basic knowledge of viral hepatitis B could be due to the hepatitis B vaccination programme organized for health workers by the Lagos state government. There was a dearth of deeper knowledge and understanding however as less than 35% were familiar with HBV immunology and the long lasting implications of resolved HBV infection. This is similar to findings from other studies done in U.S.A and Australia, among middle and high grade medical doctors; studies done in China and Iran among surgeons; and among healthcare workers in Karachi (Pakistan), where the respondents demonstrated a very low knowledge of hepatitis B infection [20–23]. This study is quite encouraging considering the fact that knowledge is usually the first step towards modification of a desirable behaviour. Modes of transmission of viral Hepatitis B include parenteral route, perinatal transmission, and sexual transmission, transmission from child-to-child (horizontal transmission) through physical contact between children with skin conditions such as impetigo and scabies or with cuts or grazes. Transmission by blood sucking arthropods is suspected but there is no convincing evidence to support this suggestion [2]. In this study, the knowledge of the respondents about the various aspects of transmission of viral hepatitis B was generally high although there was observed gap; only 34.5% of the respondents knew that a person who has recovered from viral hepatitis B does not transmit the virus. This finding is at variance with the findings from the studies carried out in USA, Australia, Pakistan, China and Iran which revealed that the knowledge about the transmission of viral hepatitis B was low [20, 21]. The low knowledge of transmission of viral Hepatitis B infection in USA, Australia, China and Iran might be because these countries are not viral Hepatitis B endemic areas. This study revealed that all the respondents knew that viral hepatitis B can be transmitted through blood transfusion, 97% knew it can be transmitted through needle stick injuries (NSIs) and 91% knew it can be transmitted through sexual intercourse. These findings are higher than the findings from a study carried out in Karachi among health care workers in a tertiary hospital where 95% of the respondents knew that viral hepatitis B can be transmitted through blood transfusion, 92% knew it can be transmitted through NSIs and 78% knew it can be transmitted through sexual intercourse [23]. The findings in our study are also higher than the findings from a study among health workers in southern Nigeria where 68.5% knew viral hepatitis B can be transmitted through NSIs and 37% knew it can be transmitted through sexual intercourse [24]. High risk groups comprise the of blood transfusions, health care and laboratory personnel, homosexuals, prostitutes, percutaneous drug abusers, infants of HBV carrier mothers and mothers who are immuno-compromised [2]. In this study, the finding that 91% of the respondents knew that healthcare workers (HCWs) are at risk is higher than the finding in Karachi study which reported that 65% of the respondents knew that all HCWs are at risk [23]. The difference might be due to the fact that the Karachi study was carried out among doctors, medical students and paramedical staff. In this study, 91% of the respondents were aware that HBV can be transmitted from patients to HCWs and 98.5% knew that the virus can be transmitted from HCWs to patients. These findings are higher than the findings in the study carried out in Kuwait among HCWs in primary health care centres where more than three quarters of the respondents (76.2%) were aware that HBV can be transmitted from patients to HCWs and 57.7% knew that the virus can be transmitted from HCWs to patients [17]. It is also higher than the finding from a study carried out among HCWs in Southern Nigeria where 80.9% of the respondents reported that viral hepatitis B can be acquired as nosocomial infection [24]. Since there is no specific curative treatment, prevention has been the major aim in managing viral hepatitis B. One of the preventive measures is administration of hepatitis B vaccine. The plasma derived vaccine is based on the surface antigen (HbsAg) which is harvested and purified from the plasma of human carriers of hepatitis B virus. The vaccine is given in 3 doses. An effective antibody response is attained after 3 doses in 95% of vaccines. In this study, 82.1% of the respondents were aware that three doses of Hepatitis B vaccine are required for complete vaccination whereas only 65.7% knew the correct interval between the doses. This is higher than the findings from a study carried out in Kuwait among HCWs in a primary health care centre which revealed that 65.9% of the respondents were aware about the number of doses of vaccination required for complete protection, whereas only 44.4% answered correctly about expected interval between the doses [17]. The finding in this study that 74.6% of the respondents knew that vaccination provides protection due to an effective antibody response which is generally attained after 3 doses in 95 percent of vaccinees is lower than the finding from the Karachi study in which 89% of the respondents believed that vaccination provides protection [23].

**Practice of Viral Hepatitis B vaccination:** in this study, 11.9%, 17.2% and 48.5% of the respondents had received 1 dose, 2 doses and complete 3 doses of HBV vaccine respectively. The finding that only 11.9% of the respondents had received only 1 dose of hepatitis B vaccine is lower than the finding of 40.6% from the study conducted in Egypt [25]. The finding that 77.6% of the respondents in this study had received at least one dose of Hepatitis B vaccine, contrasts the findings of the studies carried out in Iran (93.3%) [26], Kenya (47.5%, 12.8%) [27, 28], South Africa (21.2%) [29] and southern Nigeria (70.2%) [24]. In this study, only 29% of the respondents checked their immunity status against viral hepatitis B, this is at variance with the findings of the study conducted in Iran which reported that 56.8% had checked their antibody levels [26] and the study conducted in Kenya which revealed that none of the respondents had previously checked their immunity status against hepatitis B [28]. This is particularly important in sub-Saharan Africa where most viral Hepatitis B infections are acquired in childhood. In these places, adult vaccination especially in health care workers should be accompanied by evaluation of protective anti HBs titres post vaccination. The practice of post vaccination antibody testing noted in this study, while consistent with reports from Kenya and Pakistan, is at variance reports from Johannesburg, South Africa [29] where up to 45% of HCW performed post vaccination testing. This may be related to the socio-economic characteristics of the studied population, the test availability and the availability of publicly funded HBV treatment programme in South Africa.

## Conclusion

In conclusion, Prevalence of HBsAg was low; knowledge of basic viral hepatitis B infection was good while knowledge of HBV immunology was poor. Practice of hepatitis B vaccination was poor. It is therefore recommended that the state ministry of health should organise HBV training and education for HCWs. The ministry should also institute compulsory occupational hepatitis B vaccination programme and post vaccination anti-HBS testing to ensure adequate antibody level in this adult population.

### What is known about this topic

- Prevalence of hepatitis B surface antigen is relatively high among health care workers (doctors and nurses) in Nigeria.

### What this study adds

- We found prevalence of hepatitis B surface antigen is low among the doctors and nurses;
- We found Knowledge of HBV immunology is poor among the doctors and nurses.
